# China’s logistics evolution: A study of development characteristics and catalytic effects on economic growth

**DOI:** 10.1371/journal.pone.0309737

**Published:** 2024-09-09

**Authors:** Fuwei Qiao, Qinzhe Yang, Wei Shi, Xuedi Yang, Wei Ma, Lulu Zhao, Guanwen Ouyang

**Affiliations:** 1 College of Economics, Northwest Normal University, Lanzhou, Gansu, China; 2 College of Geography and Environmental Science, Northwest Normal University, Lanzhou, Gansu, China; 3 College Earth and Environmental Sciences, Lanzhou University, Lanzhou, Gansu, China; 4 College of Economics and Management, Huaibei Normal University, Huaibei, Anhui, China; Thammasat University Institute of East Asian Studies, THAILAND

## Abstract

The impact of logistics development on the economy covers many aspects, such as production, cost, employment, international trade, etc. It is an indispensable part of the modern economy, which helps to improve overall economic efficiency and social prosperity. This study studies the spatiotemporal dynamic evolution characteristics of China’s logistics development from 2008 to 2018 and explores its impact on economic growth in multiple dimensions of time and space. The research findings indicate the following: (1) From 2008 to 2018, China’s logistics development level (LDL) exhibited a clear upward trend. The differences between the eastern, central, and western regions showed fluctuating downward patterns, ultimately converging towards a high-level concentration. Concerning spatial distribution, China’s logistics development demonstrated a trend towards the west and south. However, the spatial pattern of "strong in the east and weak in the west " has existed for a long time. Moreover, the "T-shaped" pattern between coastal provinces and those along the Yangtze River Basin deepened, and the LDL in the central and western regions significantly improved. (2) The advancement of China’s LDL effectively promotes its economic growth, confirming that the Belt and Road Initiative enhances the role of logistics development in driving economic growth. Regarding regional differences, logistics development positively influences economic growth in the eastern and western regions, with a less significant impact on the central region. Regarding the strength of influence, logistics development has a more substantial effect on promoting high-ranking provinces in the entire country, the eastern region, and the central region, as well as boosting economic growth in low-ranking provinces in the western region.

## 1. Introduction

Logistics, as the link between various industries in a national economy, is integral to both economic production and consumption. Under the new development pattern, in which the domestic economic cycle plays a leading role while the international economic cycle remains its extension and supplement, the rapid development of logistics is undoubtedly the basis for improving China’s trade competitiveness. With the intensification of international trade competition, promoting high-quality logistics development has become crucial for countries to integrate into the global value chain and encourage economic growth [1, 2]. Relevant studies have proven that the economic development of the G7 (United States, United Kingdom, Germany, France, Japan, Italy, and Canada) results from the stimulus of logistics and government consumption [3]. The NDRC (National Development and Reform Commission) pointed out that the logistics industry is a primary, strategic, and leading industry supporting the development of the national economy.

As the world’s second-largest economy, the importance of high-quality logistics development for economic growth is evident. According to statistics, in 2021, China’s total social logistics reached 335.2 trillion yuan, 3.7 times that in 2008 [4]. To meet the challenges of the new round of scientific and technological revolution and industrial transformation, the contraction of the global industrial and supply chains, and the intensifying competition between major countries, China should enhance the breadth, depth, and resilience of its economy and trade in global competition, and realize a new development pattern with a virtuous cycle at the core of production, distribution, circulation, consumption, and other links. Therefore, from the perspective of the new development pattern, this study comprehensively evaluates the dynamic evolution characteristics of China’s logistics development in recent years and discusses its impact on economic growth in multiple dimensions. This approach is conducive to realizing the dynamic circulation of the economic cycle and unimpeded industrial correlation, achieving a dynamic balance of supply and demand, and accelerating the transformation of China’s economy to high-quality growth.

Therefore, this study measures the temporal and spatial evolutionary characteristics of China’s logistics development and its impact on economic growth. The remainder of this paper is organized as follows: Section 2 systematically reviews previous research. Section 3 summarizes the methodology used in this study, including Theil’s coefficient, kernel density estimation, the standard deviation ellipse, and sampling regression. These methods are used to evaluate the differences in the development level of logistics between regions in China, measure the development level of regional logistics in different years, analyze the spatial transfer characteristics of China’s logistics centers during the study period, estimate the impact of logistics development on economic growth, reveal the spatiotemporal evolution characteristics of China’s logistics from multiple perspectives, and explore the effects of logistics development on economic growth. Sections 4 and 5 explain the spatio-temporal dynamic evolution of China’s logistics development and its impact on economic growth, respectively. Section 6 presents conclusions and policy recommendations.

## 2. Literature review

The concept of logistics was first developed in the United States in the 1930s. The depth and breadth of logistics development vary across different stages of societal progress, leading to a changing emphasis on measuring logistics advancement. Early stage research primarily focused on qualitative analysis and functional assessment with measurements of logistics development, concentrating on the value generated by transportation activities. These activities encompass high-speed roadways, seaports, land transportation systems, aviation, and other transportation infrastructure and services. The level of logistics development is evaluated based on these factors [5, 6]. As society progresses and research deepens, transportation infrastructure has become a subsystem for measuring the development of logistics. It is integrated into a comprehensive evaluation system along with subsystems such as regional economic development, industrial conditions, and the level of informatization to measure the advancement of logistics [7]. Recently, the role of factors such as government initiatives and new economic systems in promoting regional logistics development has become increasingly important. These elements have been widely incorporated into evaluation indicator systems [8]. In addition, in the context of the Information Age, intelligence’s role in the socioeconomic landscape has grown significantly. Indicators related to the characteristics of the Information Age, such as Intelligent Logistics, have also been integrated into evaluation frameworks for logistics development. For instance, Liu et al. [9] construct an Intelligent Logistics Ecological Index to assess the current status and potential issues in the development of intelligent logistics ecosystems and chains.

The development of the logistics industry is unbalanced due to the influence of resource factor endowment and the market environment, resulting in evident spatial and temporal differences between different regions. The research scale includes all continents [10, 11], countries [12, 13], and economic belts [14]. The research mainly focuses on green logistics practices [15], reverse logistics [16], smart logistics [17, 18], logistics efficiency [19], and spatial patterns of logistics facilities [20]. The results mainly reveal the developmental stage of time and characteristics of the spatial aggregation of LDL. Pokrovskaya [21] discussed the evolution and development trends of logistics ecosystems in the context of COVID-19.

Logistics development is affected by many factors, including social and economic factors as well as natural conditions. Transportation infrastructure plays a fundamental role in logistics development. To promote economic development, the state should actively build roads, bridges, canals, and other public facilities to ensure that the development of transportation infrastructure adapt to economic development. Infrastructure construction has a significant role in promoting the development of logistics [22]. For instance, Deng [23] evaluated the relationship between ports and logistics in China’s Yangtze River Economic Belt. In addition, logistics competitiveness, the location of logistics centers, the level of information technology, freight prices, and transportation modes affect logistics development [24, 25]. For example, Kang et al. [26] studied the consumer economy, factor trade-off, external shocks, and regulatory reform as influential factors in the development of logistics in the Seoul Metropolitan Area, South Korea. Tian et al. [27] show that transportation infrastructure, regional economic conditions, informatization level, and human capital factors are positively correlated with the development level of logistics, and these factors have apparent promoting effects on the development of logistics in China. For logistics in some specific regions, the key impact factors differ. For example, market and political factors are the main factors affecting the development of port logistics in Hong Kong [28]; the information level is the key factor for the success of enterprise logistics [29]; and land use planning, industrial demand, transport supply, and urban structure are the main factors affecting the spatial evolution of logistics facilities in Shanghai [30].

Because logistics plays a vital role in economic development, research on the relationship between logistics development and regional economic development has always been a hot topic among many scholars. The goal of realizing the coordinated development of regional logistics and economy and achieving a win-win situation has been proposed [31]. For example, Wang et al. [32] discussed the long-term balance and causal relationships among nine logistics infrastructures in China, including sea transport, air transport, road transport, and economic development. Qi et al. [33] show that logistics infrastructure has a positive spatial spillover effect on regional development during different periods. Li et al. [34] showed that logistics have high spatial spillover effects on agricultural economic growth in eastern, central, and western China. Based on a network equilibrium model, Branco et al. [35] studied the economic and environmental impacts of an increase in new high-speed rail in Brazil. Amin et al. [36] studied the effect of maritime logistics on Indonesia’s economic development and found that maritime logistics costs and tariffs were essential factors restricting regional economic development. Commonly used methods are the dynamic model [37], VAR model, dynamic structure model [38], and Bayesian network analysis [31]. The prevailing view is that economics and logistics must be mutually reinforced. On the one hand, economic development determines the degree of development of logistics and then stimulates the future development of logistics [39]. However, the logistics industry’s development shifts its economy to a certain region.

Many scholars have conducted extensive research on this topic, accumulating a wealth of theoretical foundations and case data for subsequent studies. The main contributions are as follows: (1) In the measurement and evaluation of logistics development, the research method has changed from qualitative to quantitative analysis of the evaluation index system, and the evaluation system is constantly improving with the social development stage. (2) Existing studies have explored the spatiotemporal evolution characteristics of logistics development at different scales from different national, provincial, economic belts, urban agglomerations, and other perspectives, and have accumulated a wealth of typical cases. (3) In terms of research on influencing factors, many scholars have used methods such as the grey correlation degree and impulse response function spatial econometric model to reveal the degree of influence of logistics networks, transportation infrastructure, industrial structure, and other factors on logistics development, which has strong guidance for exploring factors affecting logistics development. (4) The relationship between logistics development and regional economic development has attracted the attention of many scholars. The research results are fruitful and can effectively guide the coordinated development between logistics development and the regional economy and promote high-quality economic development. Given this, this study discusses the influence of long-term time series and the difference in spatial influence between the East and West on the dimensions of time and space. Moreover, the quantile characteristics of the East and the West at different points were revealed, and the research content was more detailed and accurate. Specifically, the innovation of this study mainly focuses on the following three aspects: (1) The index system of logistics development is constructed from the perspective of a “dual circulation” development pattern, which can better reflect the comprehensive characteristics of logistics and better apply to the existing policy background in China compared with the existing research. (2) It provides an in-depth analysis of the dynamic evolution of logistics development in China’s provincial areas. (3) This study systematically evaluated the impact of logistics development on economic growth in two dimensions: time and space.

## 3. Methods and data sources

### 3.1 Establishment of index system

The index system of this study was established based on the research results of Ma et al. [40] and comprehensively measures the development of China’s logistics from 13 indicators in three dimensions: regional economic support capacity, logistics infrastructure, and logistics operation and development (Table 1). The level of regional economic development is the driving force behind logistics development, and the circulation of elements can significantly promote logistics development. The construction of the index system includes five indicators: industrial structure and investment level. Logistics infrastructure is an important guarantee for logistics development because infrastructure such as transportation infrastructure and information infrastructure are the basic conditions for realizing the spatial flow of factors. Therefore, this indicator system includes four indicators: the number of miles of transportation modes and the number of points in the postal network. Logistics operations and development reflect the current scale of regional logistics development and the essential abilities of logistics development.

**Table 1:** Comprehensive evaluation index system of logistics development level [40].

General Objective	subgoal	index	index calculation
Logistics development level (LDL)	Regional economic support	Economic development	per capita GDP
		industrial structure	Value added of the second and third industries /GDP
		Investment level	Total fixed assets investment /GDP
		Consumption level	Total retail sales of social consumer goods /GDP
		Openness	Total Export-Import Volume /GDP
	logistics infrastructure	transport infrastructure	Mileage of highways, railways, and waterways /land area
		Postal infrastructure	postal network/ population
		Internet penetration rate	Number of internet users /population
		Telephone penetration	Number of mobile phones owned/population
	Logistics operation and development	Logistics freight volume	Highway, railway, waterway, air/land freight volume
		Logistics output scale	Value added in transportation, warehousing, and postal industries/GDP
		Logistics employment scale	Employment in transportation, warehousing, and postal industries / Total Employment
		Logistics investment scale	Investment in transportation, warehousing, and post and telecommunications / Total fixed assets investment

### 3.2 Research technique

#### 3.2.1. Theil coefficient

The Theil coefficient, proposed by Dutch economist Henri Theil, is an essential indicator of regional differences. The greater the value, the greater are the regional differences. The formula is as follows [41].

$$T=\sum_{i=1}^{N}y_{i}log\frac{y_{i}}{p_{i}}$$ (1)

Where, N is the number of study areas, *y*_*i*_ is the overall proportion of the LDL index of region i, and *p*_*i*_ is the overall proportion of the population of region i.

Using the first-order decomposition of the Theil index, the total difference level in China can be decomposed into inter-regional and intra-regional differences in the eastern, central, and western regions. The Theil index for the total difference is as follows:

$$T=T_{br}+T_{wr}$$ (2)

$$T_{br}=\sum_{i=1}^{N}y_{i}\log\frac{y_{i}}{p_{i}}$$ (3)

$$T_{wr}=\sum_{i=1}^{N}y_{i}(\sum y_{ij}\log\frac{y_{i}}{p_{i}})$$ (4)

where T is the Theil coefficient, representing the overall difference in logistics development in China; T_*br*_ is the intergroup Theil index, representing the difference between the eastern, central, and western regions; T_*wr*_ is the intragroup difference, representing the difference within the three regions; N is the number of regions; *y*_*i*_ is the proportion of *i* regional LDL index in the entire region; and *p*_*i*_ is the proportion of the population of region *i* in the entire country.

#### 3.2.2. Kernel density estimation

Kernel Density Estimation is a nonparametric estimation method that studies distribution characteristics based on sample data. Its feature is that no prior knowledge of the data distribution is used, and no assumptions are made regarding the data distribution, thus ensuring the original characteristics of the data. Kernel density estimation is a process of surface interpolation through discrete sampling points, and a continuous density curve is used to describe the distribution characteristics of random variables. It is more accurate and smoother than histogram estimation and can better describe the distribution characteristics of variables. The specific formula is as follows [42]:

$$f(x)=\frac{1}{nh}\sum_{i=1}^{n}K(\frac{y_{i}-y}{h})$$ (5)

where *n* is the number of study areas, *h* is the bandwidth, *K* () is a random kernel function, weighting function, or smoothing function.

#### 3.2.3. Standard deviation ellipse

The standard deviation ellipse can better analyze the direction and distribution of the factors. Taking the level of logistics development as a weight, this study analyzes the spatial transfer characteristics of China’s logistics development from the perspective of distribution direction and centrality. The specific formula is as follows [43]:

$$\bar{X}=\sum_{j=1}^{n}w_{j}a_{j}/\sum_{j=1}^{n}w_{j}$$ (6)

$$\bar{Y}=\sum_{j=1}^{n}w_{j}b_{j}/\sum_{j=1}^{n}w_{j}$$ (7)

where $\bar{X},\bar{Y}$ is the weighted average centers, *i* represents the location data for logistics, *n* is the total number of logistics locations, *w* is the area of logistics site *j*, and a and b represent the longitude and latitude coordinates of logistics location *j*.

#### 3.2.4. Hot spot analysis

The Getis-Ord Gi* index can reflect the concentration of local high values (" hot spots ") and low values (" cold spots ") through the spatial expression of the Z-value of the scale of urban and rural settlements in Tibet. The calculation formula is as follows:

$$G^{*}(d)=\sum_{i=1}^{n}\sum_{i=1}^{n}w_{ij}(d)x_{i}/\sum_{j=1}^{n}x_{j}$$ (8)

Where *x*_*i*_ and *x*_*j*_ are the development level of logistics in the research units *i* and *j*; *w*_*ij*_*(d)* is the spatial weight of regions *i* and *j*.

#### 3.2.5. Cobb-Douglas production function

The Cobb–Douglas production function is a basic model for the quantitative analysis of factors affecting economic growth, and is expressed as:

$$Y=AL^{\alpha }K^{\beta }$$ (9)

Where *Y* is the economic output, *A* is the technical level and is a constant term, *L* is the labor input, *K* is the capital input, and *α* and *β* represent the elastic coefficients of labor input and capital input-output, respectively.

According to the neoclassical economic growth theory, technological progress affects economic output. Many scholars have expanded the production function based on the original classical Cobb-Douglas function. In this study, LDL was incorporated into the production function as a variable input factor for technology level, and a new model was obtained:

$$Y=AL^{\alpha }K^{\beta }X^{\lambda }$$ (10)

Where *X* represents logistics’ development level, λ represents logistics’ output elasticity coefficient. In the general solution process, the logarithm of both sides of Eq (3) is obtained as follows:

Ln(Y)=LnA+αLnL+βLnK+λLnX (11)

### 3.3 Data sources

This study selected data from 31 provinces in China from 2008 to 2018 for analysis; Hong Kong, Macao, and Taiwan provinces were excluded due to unavailable data. Population, per capita GDP, total fixed asset investment, total retail sales of consumer goods, and total imports and exports at the end of the year were sourced from the China Statistical Yearbook. Highway, railway, waterway mileage, number of postal outlets, added value of transportation, warehousing, and postal industry, employment in transportation, warehousing, and postal industry, and investment in transportation, warehousing, and post and telecommunications were all sourced from the China Transportation Yearbook. Other panel data were obtained from the China City Statistical Yearbook and the provincial statistical yearbook.

## 4. Spatial and temporal dynamic evolution of LDL

### 4.1 Time evolution characteristics

#### 4.1.1 Temporal change of difference

**Fig 1:**
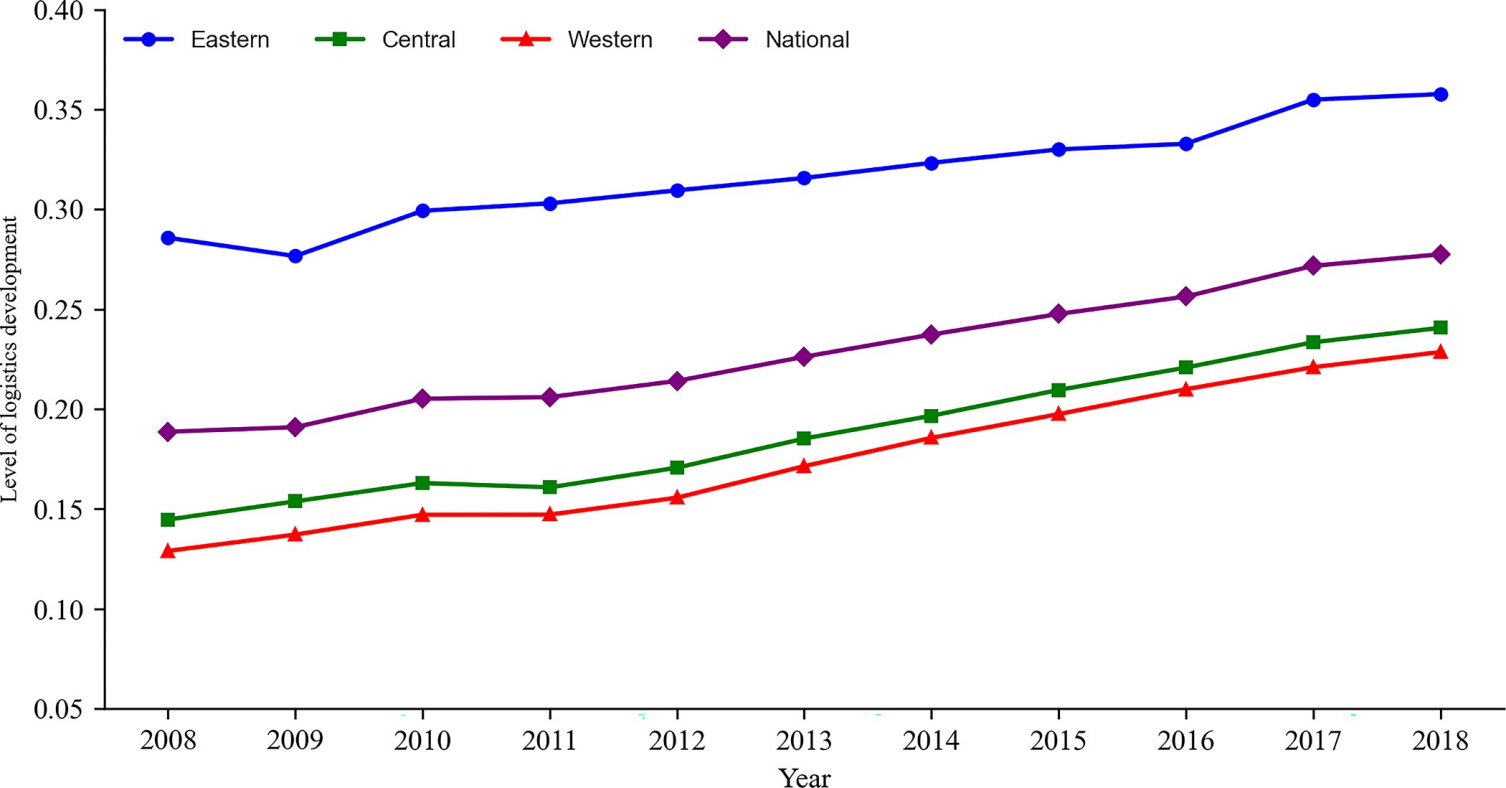
Evolution trend of China’s LDL from 2008 to 2018.

During the study period, the development level of logistics in China and the three regions of Eastern, Central and Western showed an obvious upward trend, and the pattern of "Eastern > Central > Western" existed for a long time (Fig 1). From 2008 to 2018, China’s LDL index increased from 0.189 to 0.277, and LDL increased by 46.56%. Affected by the 2008 global financial crisis, the development of China’s logistics grew slowly in 2009. However, with the adjustment of national macro policies, the implementation of proactive fiscal policies, moderately loose monetary policies, and the introduction of a series of policies to expand domestic demand, China’s economic development steadily increased, new business forms emerged in regional coordination, and China’s logistics development showed rapid and stable growth. From a regional perspective, LDL presents a pattern of east > central > west, but the growth rates in the west and central regions are significantly higher than those in the east. From 2008 to 2018, the development level of logistics in the western region increased from 0.129 to 0.229, an increase of 77.16%; in the central region, it increased from 0.145 to 0.241, an increase of 66.31%; and in the eastern region, it increased by 25.16%. The Western Development Strategy and the Belt and Road Initiative have provided good opportunities for the development of logistics in the West. The continuous improvement of infrastructure such as high-speed rail and highway networks in the central and western regions has laid the foundation for the development of logistics in these regions. The experience, lessons, and high-level spillover effects of logistics construction in the East have accelerated the development of logistics in the Middle and Western regions.

**Fig 2:**
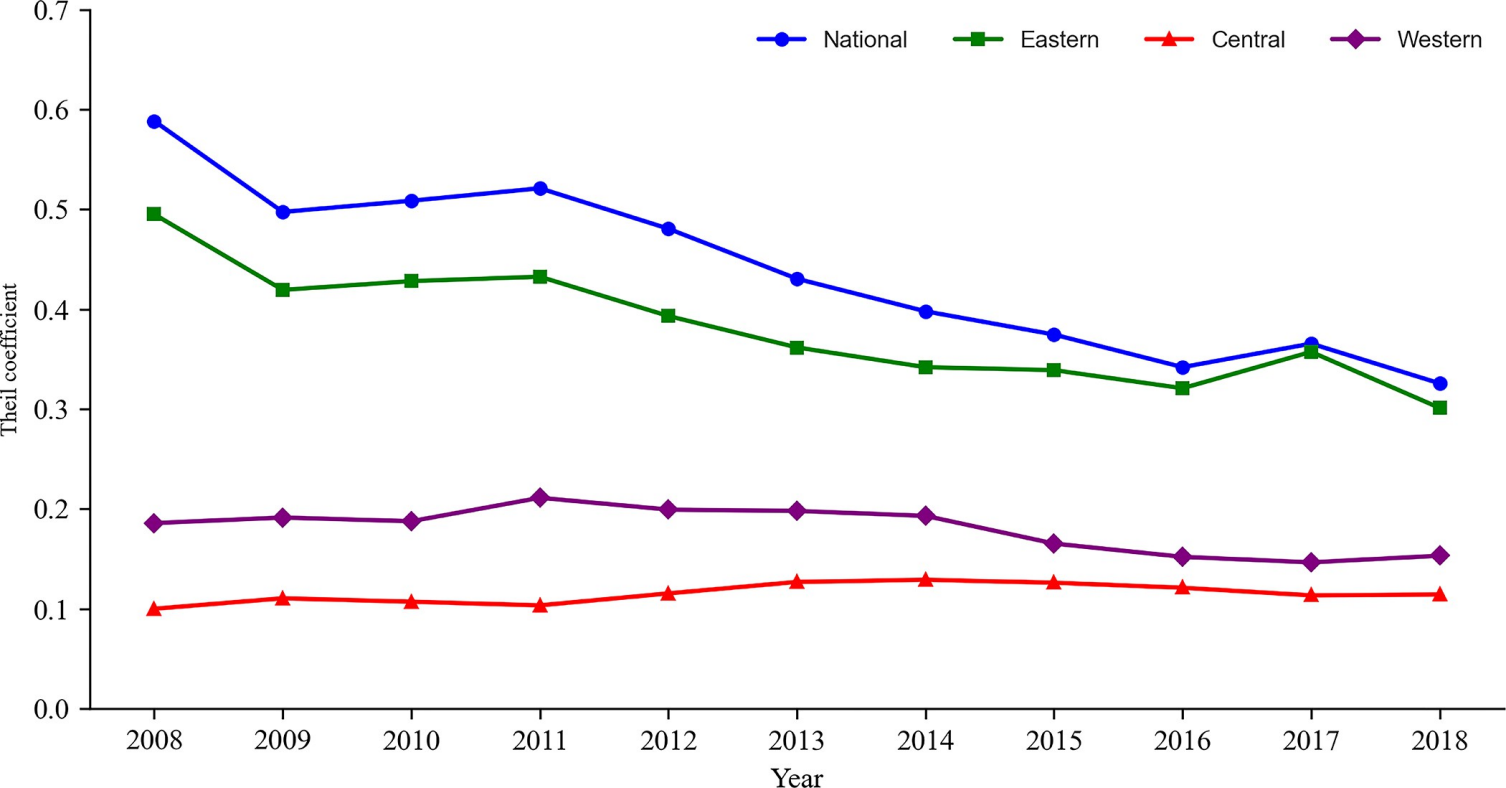
Coefficient of variation of China’s LDL from 2008 to 2018.

**Fig 3:**
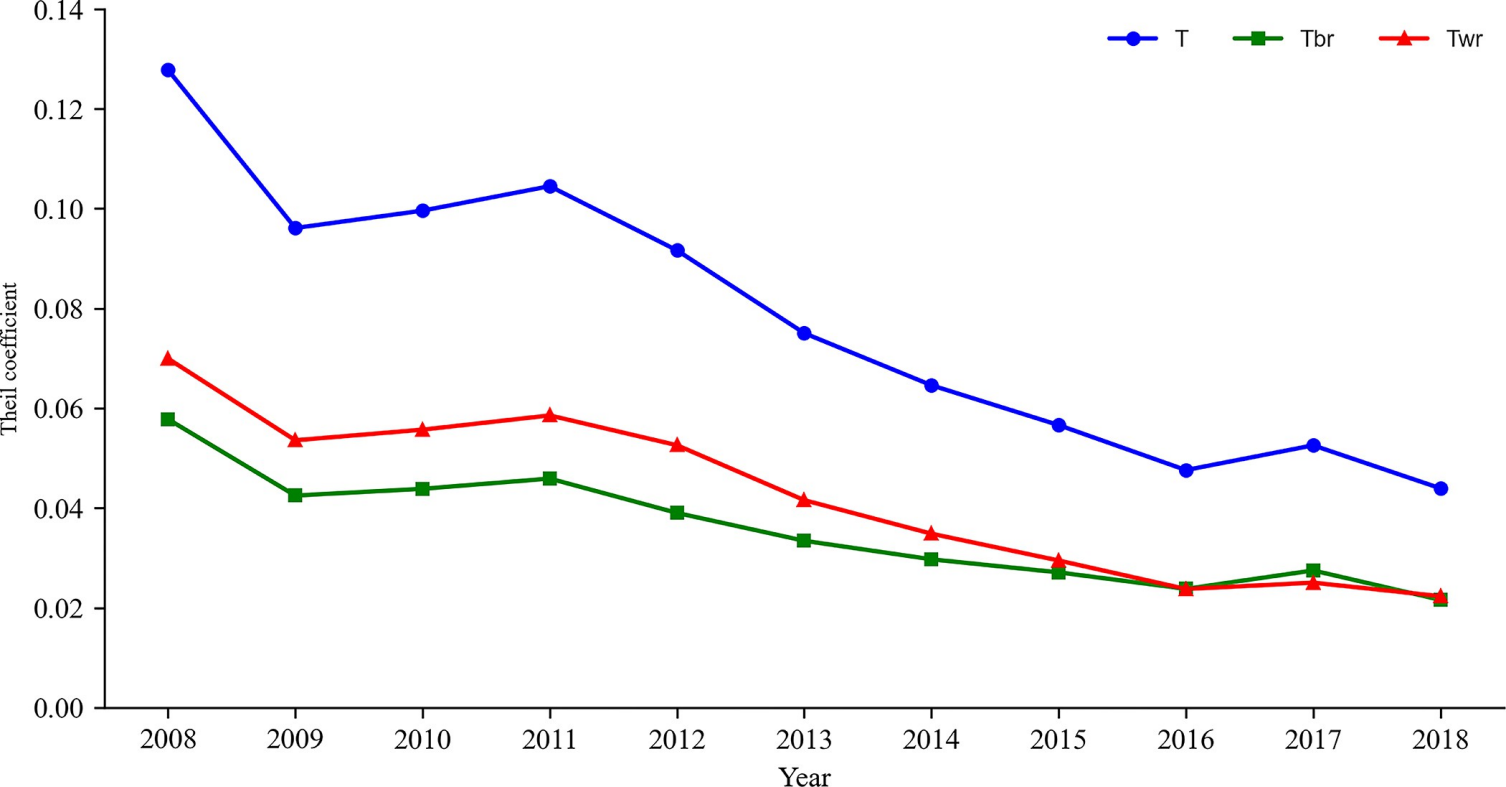
Theil coefficient of China’s LDL from 2008 to 2018.

From 2008 to 2018, regional differences in China’s logistics development show a decreasing volatility trend, with inter-regional differences being the main contributors (Figs 2 and 3). From a national perspective, from 2008 to 2018, the coefficient of variation fluctuated from 0.588 to 0.323 and the coefficient fluctuated from 0.128 to 0.044, with two anomalies of a downward trend formed in 2009 and 2017. The formation of the anomalies in 2009 may be related to the greater impact of the financial crisis on social development in the eastern region, which is China’s economic center and is more open to the outside world. The financial crisis in 2008 had a more noticeable impact on the eastern region. In response to the 2008 financial crisis, the Chinese government implemented a series of measures such as expanding government investment and promoting tax reform. After the economic recovery, the east-west gap returned to the average level. In 2017, the anomaly of the east-west gap widened, which may be mainly related to the great impact of the national supply side structural reform on the economic development of the eastern part of the country. From the perspective of difference decomposition, from 2008 to 2018, the fluctuation of the difference within the eastern region was more apparent, with the coefficient of variation decreasing from 0.495 to 0.301, while the central and western regions basically remained stable. The central region increased slightly from 0.100 to 0.114, and the western region also showed a slight reduction. The coefficient of variation decreased from 0.186 to 0.153. The results of the Thiel coefficient show that inter-regional differences are the main contributing factors to differences in China’s logistics development, and the contribution rate of regional differences is also increasing. In 2016 and 2018, the contribution rates of intra- and inter-regional differences reached 50%, indicating that with the continuous narrowing of the gap between the eastern and western regions in logistics development, the problem of eliminating regional differences must be solved urgently.

#### 4.1.2 The dynamic evolution of development level

**Fig 4:**
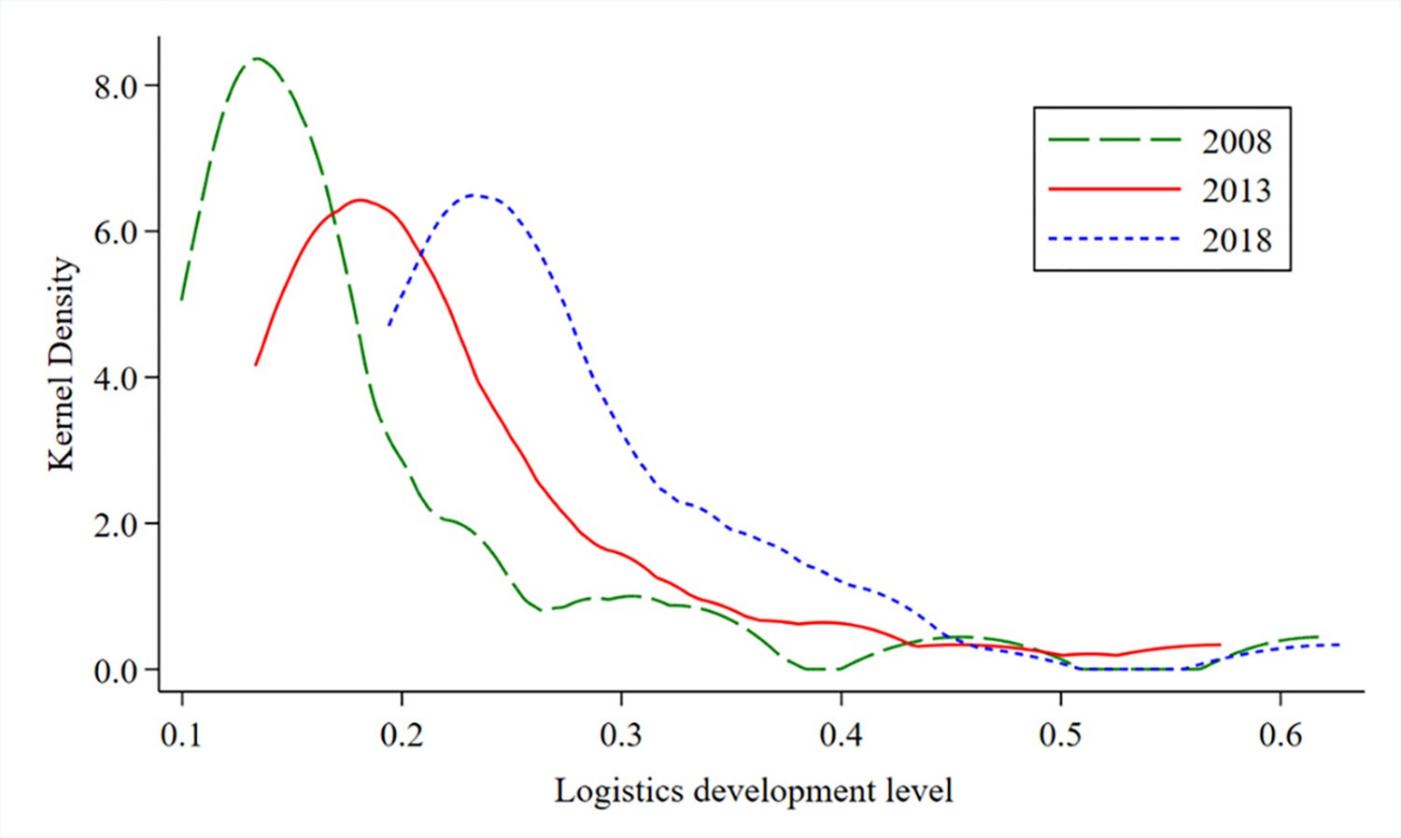
Dynamic evolution trend of China’s LDL from 2008 to 2018.

To further explore the temporal evolution of aggregate differences in China’s LDL, kernel density estimation was used to measure LDL levels in 2008, 2013, and 2019 (Fig 4). The middle line of the kernel density curve of China’s LDL shows a large right-shift trend, which indicates that China’s LDL is constantly improving, and the improvement range is large over time. The kernel density function transforms from "multi-peak" to "single-peak.” In 2008, the kernel density map showed the pattern of a main peak with multiple right-hand peaks, indicating that the development level of China’s logistics was obviously multipolar in 2008. In 2013 and 2018, it exhibited a "single-peak form,” indicating that the development of China’s logistics industry converged toward the equilibrium point. The gap between the provinces narrowed. From 2008 to 2018, the area on the left side of the wave crest showed a significant shrinking trend, indicating that the development level of China’s logistics increased in a high direction. In short, from 2008 to 2018, China’s logistics development showed a narrowing gap between high and low levels and a gradual evolution trend toward high-level agglomeration.

### 4.2 Spatial evolution

#### 4.2.1 Spatial evolution of development level

**Fig 5:**
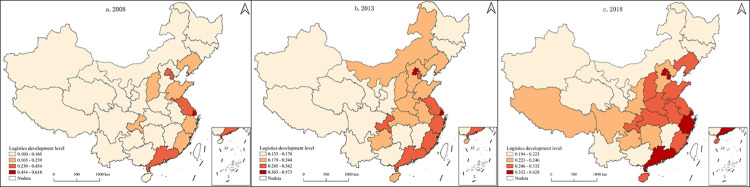
Spatial evolution of China’s LDL from 2008 to 2018. Based on the standard map GS (2019)1835 of the Ministry of Natural Resources of China service website, the map boundary is not modified during QGIS production.

To reveal the spatial evolution characteristics of LDL more clearly, this study used Jenks’ natural breaks to divide China’s provincial LDL into four types: highest, higher, lower, and lowest, and performed spatial visualization expressions. (Fig 5).

The spatial evolutionary characteristics of China’s provincial LDL were vital to the east and weak in the west. With the passage of time, the "T" structure of logistics development is becoming more and more apparent. In 2008, the overall level of logistics development in China was low, with 26 provinces having the lowest levels, accounting for 83.87% of the total. In terms of spatial distribution, the highest level was mainly distributed in the eastern coastal provinces, and the central and western provinces, except for Chongqing, all had low-level types. By 2013, the five lowest-level provinces had transformed into lower-level types, and one highest-level province had increased. Regarding spatial distribution, the development level of logistics in central China increased, and the development level of logistics in Hubei and Anhui provinces located in the Yangtze River Economic Belt improved, and a "T" type structure initially took shape. In 2018, the number of provinces at the highest and highest levels increased significantly, with the number growing from five in 2008 to nine, and the total share grew from 16.13% to 29.03%. In terms of spatial distribution, The "T"-shaped structure of logistics development has been further strengthened, with the eastern coastal provinces showing higher and highest levels, while Shaanxi and Henan in central, and Sichuan, Tibet and Guizhou in western China have all been upgraded.

#### 4.2.2 Center of gravity transfer locus

**Fig 6:**
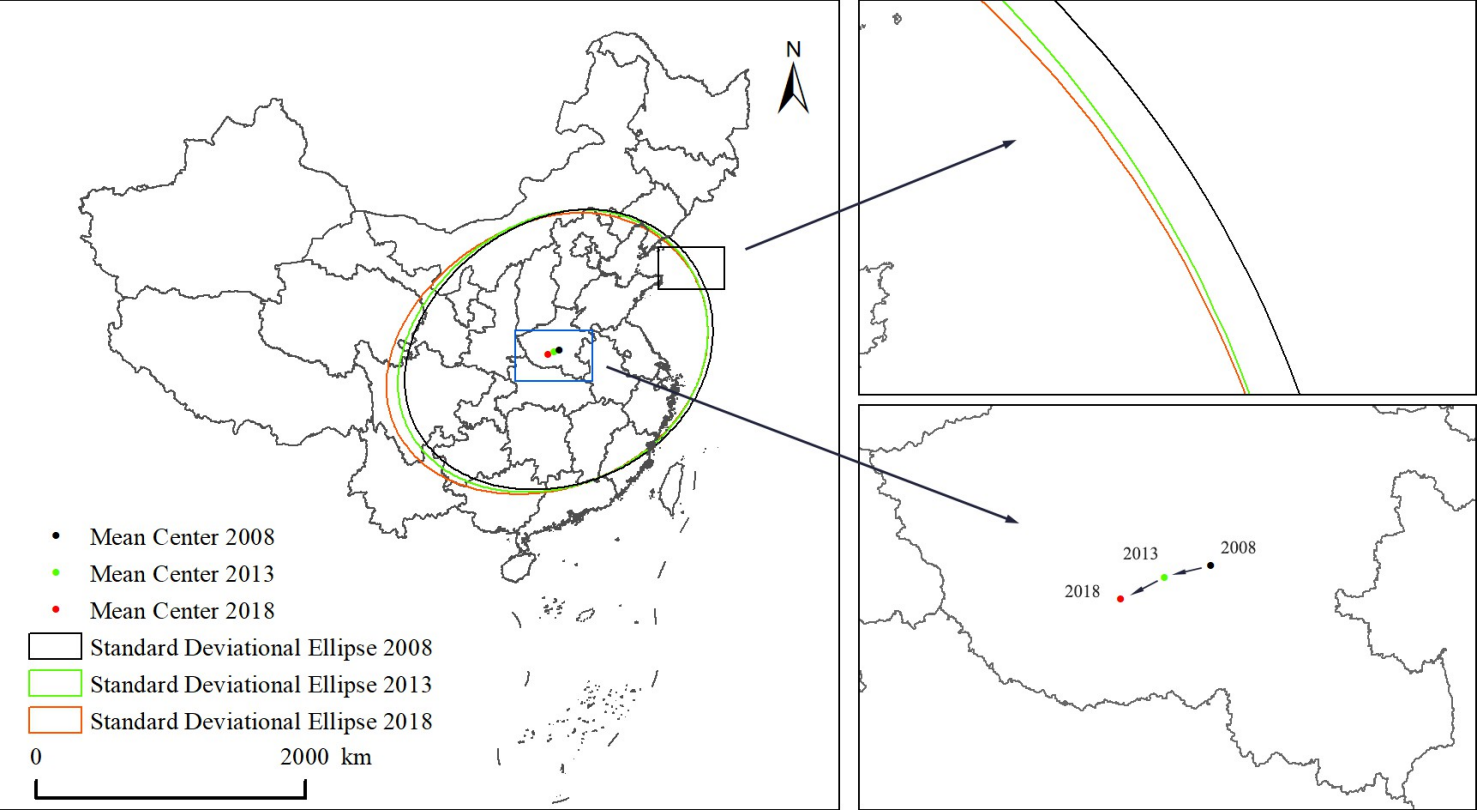
Standard deviation ellipse of China’s LDL and locus of the center of gravity migration. Based on the standard map GS (2019)1835 of the Ministry of Natural Resources of China service website, the map boundary is not modified during QGIS production.

A center-of-gravity shift trajectory was drawn to explore the spatial evolution of the center of gravity of China’s LDL (Fig 6, Table 2). From the perspective of the spatial shift of the center of gravity, the center of gravity of China’s logistics development showed a trend toward the southwest, but was always distributed in the region east of China’s geometric center, indicating that the pattern of China’s LDL in the strong east and weak west has existed for a long time, and the discrepancy between the east and west directions has slowed. From 2008 to 2018, China’s logistics center of gravity moved 89.183 km to the southwest, including 82.831 km to the west and 33.055 km to the south, indicating that the growth rate of the level of logistics development in the central and western parts of China is quite obvious and that the difference between the development of logistics in the north and south directions is relatively small. The direction angle of the standard deviation ellipse ranges from 74.82° to 75.97°, with an overall distribution pattern in the northeast-southwest direction, which corresponds to an imbalance in the development of southeast and northwest China, as represented by the two sides of the Aihui-Tengchong Line. The flat rate increases slightly from 0.405 to 0.458, indicating that the spatial gap between Northeast and Southwest China’s logistics development has increased to a certain extent. The long axis increases continuously, while the short axis increases and then decreases; however, the magnitude of both changes is very small, indicating that the decentralization trend of China’s logistics development has begun to appear.

**Table 2:** Parameters of standard deviation ellipse of logistics development in China from 2008 to 2018.

Year	Longitude of the center point	Center latitude	Long axis	Short axis	Direction angle	Oblateness
2008	113.687	33.447	12.460	8.867	75.463	0.405
2013	113.222	33.379	12.541	8.923	74.821	0.406
2018	112.770	33.235	12.975	8.899	75.969	0.458

## 5. The impact of logistics development on economic growth

### 5.1 Total estimate

Benchmark regression, spatial subsampling regression, and temporal subsampling regression were used in China’s logistics development from 2008 to 2018 using stata18 software [44]. Models 1–3 are baseline regression analyses, which mainly consider the basic regression analysis of the level of logistics development on economic growth. Model 1 uses the ordinary least squares method, which examines the impact of China’s logistics on economic growth. In addition to capital and labor based on the Cobb–Douglas production function, Models 2 and 3 examine the impact of the development level of core element logistics on economic growth when only a single factor of logistics is added and multiple factors of logistics, industrial structure, and urbanization rate are added. Models 4 and 5 show the temporal regressions. In 2013, the Belt and Road Initiative (BRI) was proposed, which injected new vitality into the development of the world economy, played a supporting and demonstration role in the modern domestic logistics structure, and is conducive to stimulating the development and upgrading of China’s logistics industry [45, 46], It is even more conducive to promoting Poverty reduction in countries along the route [45]. Therefore, Models 4 and 5 represent the temporal variation characteristics between 2008–2013 (before the Belt and Road Initiative) and 2013–2018 (after the Belt and Road Initiative), respectively. Models 6–8 are spatial sampling regressions. Affected by factors such as economy, productivity, infrastructure, marketization, informatization, and demand, the logistics industry has developed rapidly in the east and slowly in the central and western regions [47]. Therefore, Models 6, 7, and 8 explore spatial differences in the economic impact of logistics in Eastern, Central, and Western China. According to the empirical results in Table 3, the coefficients of the influence of LDL on economic growth are positive. Except for the central region, which is insignificant, all other groups pass the significance test of 1%, indicating that an improvement in LDL can effectively promote economic development.

**Table 3:** Results of the regression model.

Variable	Model 1	Mode l2	Model 3	Model 4	Model 5	Model 6	Model 7	Model 8
lnLog	1.418 ***	0.740 ***	0.380***	0.408***	0.526***	0.648***	0.033	0.241***
lnLabor		0.540 ***	0.516***	0.450***	0.599***	0.476***	0.524***	0.455***
lnInvest		0.498***	0.5286***	0.576***	0.450***	0.570***	0.611***	0.547***
lnIndustry			0.073***	0.030	0.059***	0.064**	0.120**	-0.090
lnUrban			0.332***	0.237***	0.392***	-0.130	0.404**	0.416***
Constant	11.695***	3.624***	1.51***	1.837***	1.872***	3.616***	-0.262	1.146***
*R* ^*2*^	0.269	0.976	0.980	0.982	0.982	0.970	0.917	0.9874

Note: *, **, *** indicate that the P-value passes the significance test of 10%, 5%, and 1%, respectively.

The results of the benchmark regression models 1, 2, and 3 show that the regression coefficients of logistics on economic growth are 1.418, 0.740, and 0.380, respectively, indicating that logistics development promotes economic growth positively. Through the R^2^ comparison of models 1–3, it was found that the increase in the four control variables, labor force, foreign direct investment, industrial structure, and urbanization rate, significantly improves the fit degree of the model, which can better show that the model is consistent with reality [48]. Therefore, labor force, foreign direct investment, industrial structure, and urbanization rate were added as control variables when the temporal and spatial regressions were set.

The results of time-divided regression Models 4 and 5 show that the regression coefficients of logistics on economic growth are 0.408 and 0.526, respectively, increasing by 0.118, indicating that the BRI proposal of the Belt and Road Initiative has enhanced the promotional role of logistics development on economic growth. One possible reason for this is that the international financial crisis broke out in 2008. To cope with this situation, the Chinese government planned to complete four trillion yuan of investment in ten measures to further expand domestic demand and promote steady and rapid economic growth. With the continuous stimulation of the "four trillion investment" in 2008, the growth rate of infrastructure investment went straight up, and the development of China’s logistics showed rapid growth. This provides a good foundation for subsequent economic development and effectively promotes economic growth. On the other hand, The Belt and Road" Initiative in 2013 created a broad space for the development of China’s international logistics, driving the circulation between the production, distribution, consumption, and circulation of goods involved in the domestic and international double cycles, increasing the demand for logistics, reducing logistics costs, and promoting the transformation and upgrading of the logistics industry, effectively promoting the increase in investment and exports. This provided a more decisive impetus for economic growth.

The results of spatial sampling regression models 6, 7, and 8 show that the regression coefficient of logistics development in the eastern economy is 0.648 and that in the western economy is 0.241, both of which pass the 1% significance test. The regression coefficient of the central region is positive but not significant, indicating that China’s logistics development had a positive promoting effect on both the eastern and western economies during the study period. The impact on the development of the central economy is either insignificant or not yet reflected. This is mainly due to the influence of resource endowment, location conditions, economic size, population, technology, and other aspects; transportation infrastructure development in the eastern provinces has absolute advantages. The development of logistics has led to the rapid agglomeration of capital, human capital, and other factors, resulting in scale effects and more effectively promoting circulation among the production, transportation, and consumption of commodities, the biggest boost to economic growth. Since the implementation of the Western Development Strategy in 2000, logistics in the central and western regions have developed to a certain extent, but its driving effect on the economy has not been clearly reflected.

### 5.2 Quantile regression

Concerning the research results of Wang [49] and Lin [50] et al., this study discusses the influence of LDL on the economic level in different development stages at quantiles of 0.1, 0.25, 0.50, 0.75 and 0.95 quantiles by using the basic model. Table 4 shows the regression estimation results of the panel quantile model that influences economic development factors.

**Table 4:** Regression estimation results of panel quantile model of influencing factors of economic development.

Influence factor		Quantile				
		10^th^	25^th^	50^th^	75^th^	90^th^
Nationwide	lnLog	1.869***	1.959***	2.121***	2.293***	2.425***
	lnLabor	0.423**	0.401***	0.360***	0.317**	0.283
	lnInvest	0.180	0.135	0.054	-0.033	-0.099
East	lnLog	1.383***	1.402***	1.424***	1.449***	1.474***
	lnLabor	0.019	0.001	-0.020	-0.044	-0.067
	lnInvest	0.113	0.081	0.042	-0.001	-0.044
Central	lnLog	1.127**	1.135***	1.149***	1.160***	1.168**
	lnLabor	0.404	0.401	0.396	0.392	0.388
	lnInvest	0.013	-0.094	-0.262	-0.388	-0.492
West	lnLog	2.087***	2.085***	2.079***	2.076***	2.071***
	lnLabor	0.831	0.935**	1.150***	1.294***	1.461**
	lnInvest	-0.089	-0.180	-0.368	-0.494	-0.639

Note: Values in parentheses are t-test values; ***, ** and * represent significant model results at 1%, 5% and 10% confidence levels, respectively.

According to the regression results, the panel quantile regression estimation coefficient passed the significance test at all five subpoints, indicating that logistics development plays a positive role in promoting economic development. However, in terms of the size of the regression coefficient at the national level, the eastern and central regions showed a gradually increasing trend at 0.1, 0.25, 0.50, 0.75 and 0.95 points, indicating that logistics development had a more substantial promoting effect on the top provinces in the national, eastern, and central regions. In contrast, the regression coefficients of the western region are all positive and show a downward trend, indicating that logistics development has a stronger promoting effect on low-economic-percentile provinces in the western region. A possible reason for this is the relatively low level of logistics development in the western underdeveloped areas, which are relatively isolated and have a weak ability and scope of economic activities connected with the region. With improvements in logistics, the impact on provinces with low economic development was more intense.

## 6. Conclusions and policy implications

Based on a “dual circulation” development pattern proposed by China, this study constructs an indicator system of provincial logistics development in China and analyzes the spatiotemporal dynamic evolution pattern of logistics development in China and its impact on economic growth by using models such as kernel density estimation, standard deviation ellipse, and C-D function. The main conclusions are as follows.

1. During the study period, the development level of logistics in China and the eastern, western, and eastern regions showed an obvious upward trend. However, it still maintained the pattern of "Eastern > Central > Western". China’s LDL index rose from 0.189 to 0.277, an increase of 46.56%. The difference fluctuation in the eastern region is more obvious, the coefficient of variation decreases from 0.495 to 0.301, and the change in the central and western regions is relatively small; the central region increases from 0.100 to 0.114, while the western region decreases from 0.186 to 0.153.
2. The spatial pattern of China’s LDL shows that the east is stronger than the west, and gradually forms a "T" pattern, that is, the development of logistics in coastal areas and Yangtze River basin provinces is more concentrated, while the inland areas are relatively weak. The center of logistics development moves southwest. During the study period, China’s logistics center of gravity moved 89.183 km to the southwest, including 82.831 km to the west and 33.055 km to the south. indicating the trend of China’s logistics development to the west and south, but it is still distributed in the area east of the geometric center of eastern China.
3. When analyzing the impact of logistics development and economic growth, this study finds that logistics development has a significant positive promoting effect on economic growth. A comprehensive logistics industry can help the healthy development of other real industries and provide a solid foundation for economic growth, and the proposal of the Belt and Road Initiative has enhanced the promoting effect of logistics development on economic growth. During the study period, all provinces in China achieved rapid economic growth due to the correct policy direction, prosperity, and development of various industries. As a strategic industry for optimizing industrial organizations and enhancing industrial value, logistics plays an irreplaceable role in realizing high-speed economic growth. To further accelerate the high-quality transformation of the economy, it is necessary to coordinate the links between different industries. Consolidating the basic position of the logistics industry in the national economy. The spatial sampling regression shows that the impact of logistics on the economies of different regions is different, with the eastern region benefiting more, followed by the central region, and then the western region gradually weakening. In addition, quantile regression analysis shows that logistics development has a stronger impact on the economic growth of the country, with the eastern and central provinces having the highest scores, whereas the promotion effect on the western provinces with the lowest scores is more obvious.

Based on the above conclusions, this study propose the following policy implications: First, logistics development in the western region must be strengthened. Given the fluctuating and declining trend of logistics development in the three regions of China, East and West, and the spatial pattern of "strong east and weak west,” the government can introduce logistics development policies for the western region, according to the unique geographical environment of each province in the west, explore the logistics operation mode that accords with its own characteristics, and strengthen the logistics link between cities in the west; At the same time, promote the development of western logistics industry cluster, optimize the industrial layout, and maximize the healthy role of logistics industry in economic development. Strengthen the construction of logistics infrastructure in the western and central regions, and improve the level of logistics development in the western region. To narrow the gap with the eastern region, it can realize the organic integration of logistics and resource advantages in the central and western regions and further promote the balanced development of China’s economy.

Second, China need to strengthen the cooperation between coastal and inland areas. The strengthening of the "T" pattern indicates that the high-level development of logistics in coastal areas and Yangtze River basin provinces is more concentrated, which provides an important support for the coordinated development of the central and western economies. The government can encourage cooperation between the coastal and inland areas, expand the existing logistics network in the coastal areas to the inland areas, integrate the inland areas into a wider logistics network, improve the logistics connectivity in the inland areas, promote the improvement of the logistics network, and achieve mutual benefits and win-win results.

Finally, China must continue promoting high-quality development under the Belt and Road Initiative. The results indicate that the Road Initiative can enhance the role of logistics in promoting economic growth. The Belt and Road Initiative has opened new directions for international cooperation and tapped new drivers for world economic growth. It has promoted trade between China and other countries along routes, connected domestic and international markets, and achieved mutual benefits and win-win results. The government should continue to support and promote this initiative, as well as transnational logistics cooperation, international logistics networks, and regional economic development.
